# Wing morphometric variability in *Aedes aegypti* (Diptera: Culicidae) from different urban built environments

**DOI:** 10.1186/s13071-018-3154-4

**Published:** 2018-10-26

**Authors:** Ramon Wilk-da-Silva, Morgana Michele Cavalcanti de Souza Leal Diniz, Mauro Toledo Marrelli, André Barretto Bruno Wilke

**Affiliations:** 1 Departamento de Epidemiologia, Faculdade de Saúde Pública, Universidade de São Paulo, São Paulo, Brazil; 2 Department of Public Health Sciences, Miller School of Medicine, University of Miami, Miami, FL USA

**Keywords:** Wing geometric morphometrics, Urbanization, Dengue, *Aedes aegypti*

## Abstract

**Background:**

*Aedes aegypti* is the main vector of the dengue, Zika and several other arboviruses. It is highly adapted to urbanized environments and can be found worldwide. Mosquito population control is considered the best strategy for fighting mosquito-borne diseases, making an understanding of their population dynamics vital for the development of more effective vector control programs. This study therefore sought to investigate how different levels of urbanization affect *Aedes aegypti* populations and modulate population structure in this species with the aid of wing geometric morphometrics.

**Methods:**

Specimens were collected from eleven locations in three areas with distinct levels of urbanization in the city of São Paulo, Brazil: conserved, intermediate and urbanized. The right wings of female mosquitoes collected were removed, and photographed and digitized. Canonical variate analysis and Mahalanobis distance were used to investigate the degree of wing-shape dissimilarity among populations. Thin-plate splines were calculated by regression analysis of Canonical Variation Analysis scores against wing-shape variation, and a cross-validated reclassification was performed for each individual; a neighbor-joining tree was then constructed.

**Results:**

Metapopulation and individual population analysis showed a clear segregation pattern in the Canonical Variation Analysis. Pairwise cross-validated reclassification yielded relatively high scores considering the microgeographical scale of the study and the fact that the study populations belong to the same species. The neighbor-joining tree showed that mosquitoes in the intermediate urban area segregated in the metapopulation and individual population analyses. Our findings show significant population structuring in *Aedes aegypti* mosquitoes in the areas studied. This is related to the different degrees of urbanization in the areas where the specimens were collected along with their geographical location.

**Conclusions:**

Urbanization processes in the study areas appear to play an important role in microevolutionary processes triggered by man-made modifications in the environment, resulting in a previously unknown population structuring pattern of major epidemiological importance.

**Electronic supplementary material:**

The online version of this article (10.1186/s13071-018-3154-4) contains supplementary material, which is available to authorized users.

## Background

Dengue fever is considered the most common mosquito-borne disease in the world. An estimated 3.9 billion people are at risk of infection, and there have been 390 million cases reported annually, primarily in tropical and subtropical regions [1–3]. The main vector of the disease is *Aedes* (*Stegomyia*) *aegypti* (Linnaeus), a mosquito with a worldwide distribution that is highly adapted to urbanized environments [4, 5]. It is also responsible for transmission of several other epidemiologically important arboviruses such as yellow fever virus, chikungunya virus and Zika virus [6–12].

*Aedes aegypti* can complete its entire life-cycle within a single household, using artificial breeding sites and blood-feeding on human hosts [13–15]. Moreover, man-made changes to the environment benefit it, as an increase in population density and the number of artificial breeding sites have been shown to be directly associated with a high abundance of this mosquito in urban areas [16–19]. The great abundance of *Ae. aegypti* mosquitoes, together with the multiple blood-feeding behavior of females, tend to increase host-vector interaction, in turn increasing the dissemination of pathogens [20, 21].

Mosquito population control is considered the best strategy for fighting mosquito-borne diseases, making an understanding of *Ae. aegypti* population dynamics vital for the development of more effective vector control programs. Population genetics studies provide important information about how mosquitoes react to selective pressures. One technique used in this type of study is wing geometric morphometrics based on quantitative analysis of wing venation characters, which has proved sensitive enough to detect fine-scale structuring on a microgeographical scale [22, 23]. The technique has been widely used in taxonomic and phylogenetic studies on micro- and macrogeographical scales [24–28].

Several studies using wing geometric morphometrics indicate that urbanization processes may have been modulating mosquito population dynamics [22, 25, 28, 29], leading to population structuring. This is clearly relevant to disease epidemiology, since population structuring can influence the dynamics of disease transmission. As variation in wing shape is modulated by genes, and is therefore less susceptible to selective pressures when compared with variation in wing size [30], geometric morphometric analysis of wing shape can be used to study mosquito population dynamics.

Our hypothesis is that *Ae. aegypti* is adapting locally to different urban build environments, and that wing geometric morphometrics may be able to provide important information about the population dynamics and population structuring of this species. The present study sought to use this technique to investigate how *Ae. aegypti* populations are structured in the city of São Paulo and how their interaction with areas with different levels of urbanization can modulate this structure [31–33].

## Methods

### Specimen collection

**Fig. 1:**
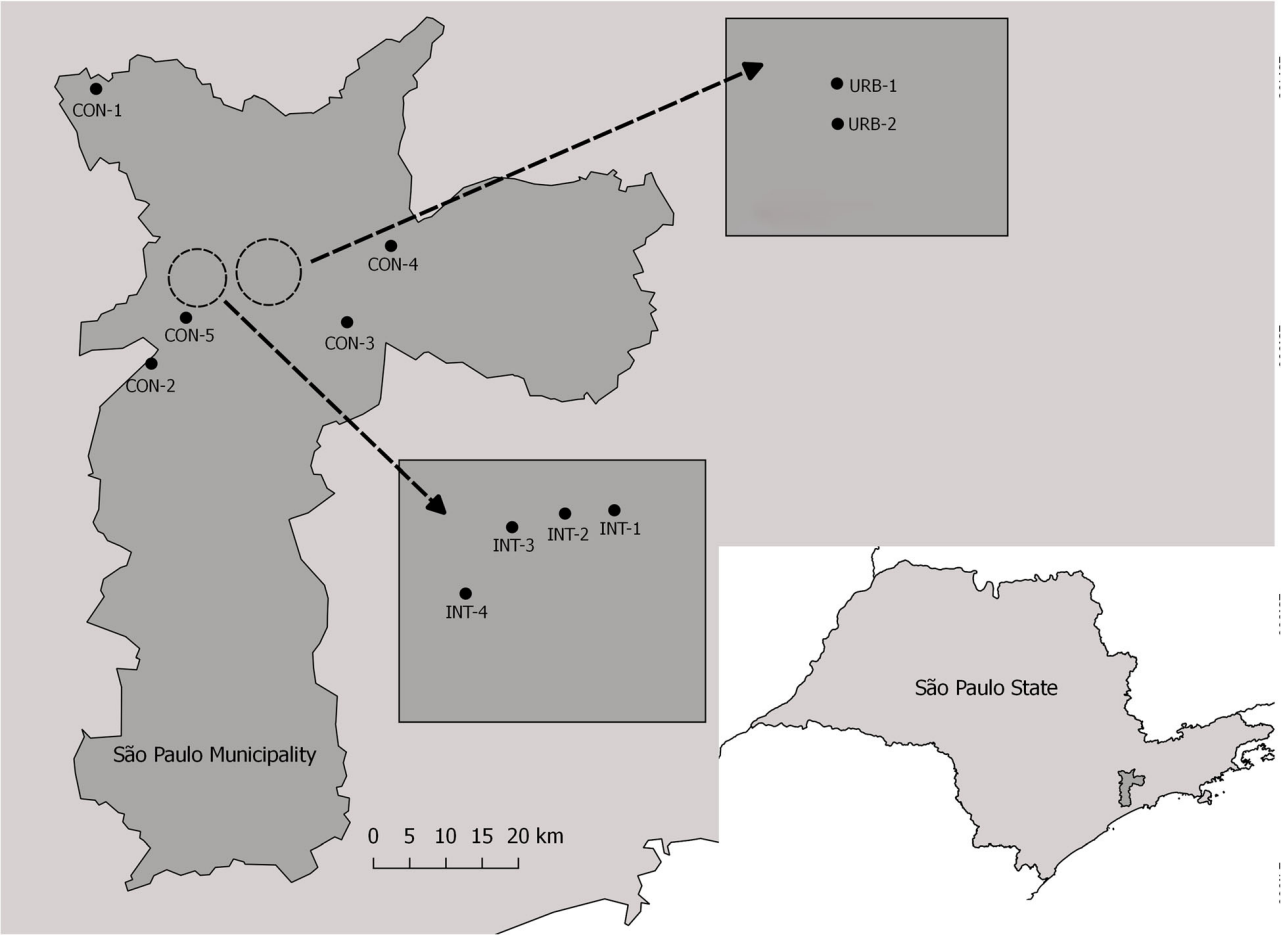
*Aedes aegypti* sampling locations in the city of São Paulo, Brazil

*Aedes aegypti* mosquitoes were collected in eleven areas, no more than 30 km apart with three distinct levels of urbanization in the city of São Paulo, Brazil (Fig. 1, Table 1).

**Table 1:** *Aedes aegypti* collection sites and collection data

Collection site	Code	Coordinates	Stage	*n*	Year
Anhanguera Park	CON-1	23°24'54"S, 46°47'6"W	Immature	13	2013
Eucalipto Park	CON-2	23°36'54"S, 46°45'18"W	Immature	30	2012
Independência Park	CON-3	26°35'6"S, 46°36'18"W	Immature	26	2015
Piqueri Park	CON-4	23°31'30"S, 46°35'30"W	Immature	30	2013
Previdência Park	CON-5	23°35'6"S, 46°43'30"W	Adult	30	2015
University of São Paulo Student Accommodation	INT-1	23°33'18"S, 46°43'30"W	Immature	30	2014
Communication and Art School	INT-2	23°33'18"S, 46°43'30"W	Immature	29	2014
Physics Institute	INT-3	23°33'54"S, 46°44'6"W	Immature	30	2014
Veterinary School	INT-4	23°33'54"S, 46°44'6"W	Immature	29	2014
Public Health School	URB-1	23°33'18"S, 46°40'30"W	Immature	30	2015
Medicine School	URB-2	23°33'18"S, 46°40'30"W	Immature	30	2015

*Abbreviation*: *n* number of specimens used

Conserved areas (CON): five municipal parks (large green areas) open to the public from 5:00 to 20:00 h. No chemical or biological control measures are used in the parks. The wild fauna includes birds, reptiles and mammals.

Intermediate areas (INT): four collection sites on the University of São Paulo Armando de Salles Oliveira campus. The campus covers 3,648,944.40 m^2^, of which approximately 800,000 m^2^ has been built on. More than 100,000 people visit, pass through or study or work on the campus every day.

Urbanized areas (URB): two collection sites on the University of São Paulo health sciences campus. This is in a highly urbanized, densely populated area and extends over 83,050.82 m^2^, of which 79,923.72 m^2^ has been built on (Table 1).

Mosquitoes were collected between 2012 and 2015. Adult specimens were collected with portable battery-powered aspirators [34], and immature mosquitoes were collected using different collection tools (Additional file 1: Table S1). In the areas where the ovitraps were used, traps were put in shaded areas at least 50 meters apart, each with 500 ml of water and hay infusion. The immature forms were kept under laboratory conditions and fed with fish food (Tetra BettaMin, Melle, Germany) until they reached adulthood. All mosquitoes collected were identified using taxonomic keys by Forattini [14] and stored in silica gel until the wings were removed. For this study, all specimens were collected during larval stages except for the population collected at Previdencia Park (CON-5), in which only adult mosquitoes were collected. Specimens were randomly selected to avoid testing siblings.

### Wing preparation and data acquisition

The right wing of each female mosquito was removed, mounted on a microscope slide with a cover slip and photographed at a magnification of 45× with a Leica DFC320 digital camera coupled to a Leica S6 microscope. Eighteen landmarks were digitized on each wing image by one of the authors (RWS) using TpsDig (v.1.40) [35], following Louise et al. [22]. The selection of landmarks was based on wing venation intersection as follows: 1st, intersections of veins Radius + Radial sector; 2nd, Costa + Sub-costa; 3rd, Radius 1; 4th, Radius 2; 5th, Radius 3; 6th, Radius 4+5; 7th, Media 1; 8th, Media 2; 9th, Media 3+4; 10th, Cubitus anterior; 11th, Mediocubital crossvein + Cubital anterior; 12th, Mediocubital + Media 3+4; 13th, Media + Media 1+2; 14th, Radiomedial crossvein + Media 1+2; 15th, Radiomedial crossvein + Radius 4+5; 16th, Radial sector + Radius 2+3; 17th, Radius 2+3 + Radius 2 and 3; 18th, Media 1+2 + Media 1 and 2 [36].

### Geometric morphometric analysis

Procrustes coordinates referent to wing shape were obtained through the superimposition of landmark coordinates. Multivariate regression of the Procrustes coordinates against centroid size (10,000 randomizations) was used to assess the allometric influence of wing size on wing shape. Canonical variate analysis (CVA) was applied and the Mahalanobis distances calculated to investigate the degree of dissimilarity in wing shape between the study populations. To visualize shape disparity, thin-plate splines were generated by regression analysis of CVA scores against wing-shape variation. Cross-validated reclassification based on the Mahalanobis distance was carried out for each individual, consisting on the blinded reclassification of each specimen taking into account only the variations in wing shape, thereby making it possible to identify levels of similarity in wing shape between populations. A Neighbor-Joining (NJ) tree was constructed with 1000 bootstrap replicates to illustrate the patterns of variation among the populations (27 specimens of *Ae*. *albopictus* were used as outgroup), and an UPGMA dendrogram was also constructed based on geographical distances between collection sites (Additional file 2: Figure S2). The analyses were carried out and graphs plotted with MorphoJ (v.2.0) [37] and PAST (v.3.16) [38]. A linear correlation analyses between Procrustes values and geographical distance (km) was performed using PAST (v.3.16).

## Results

### Wing shape

**Fig. 2:**
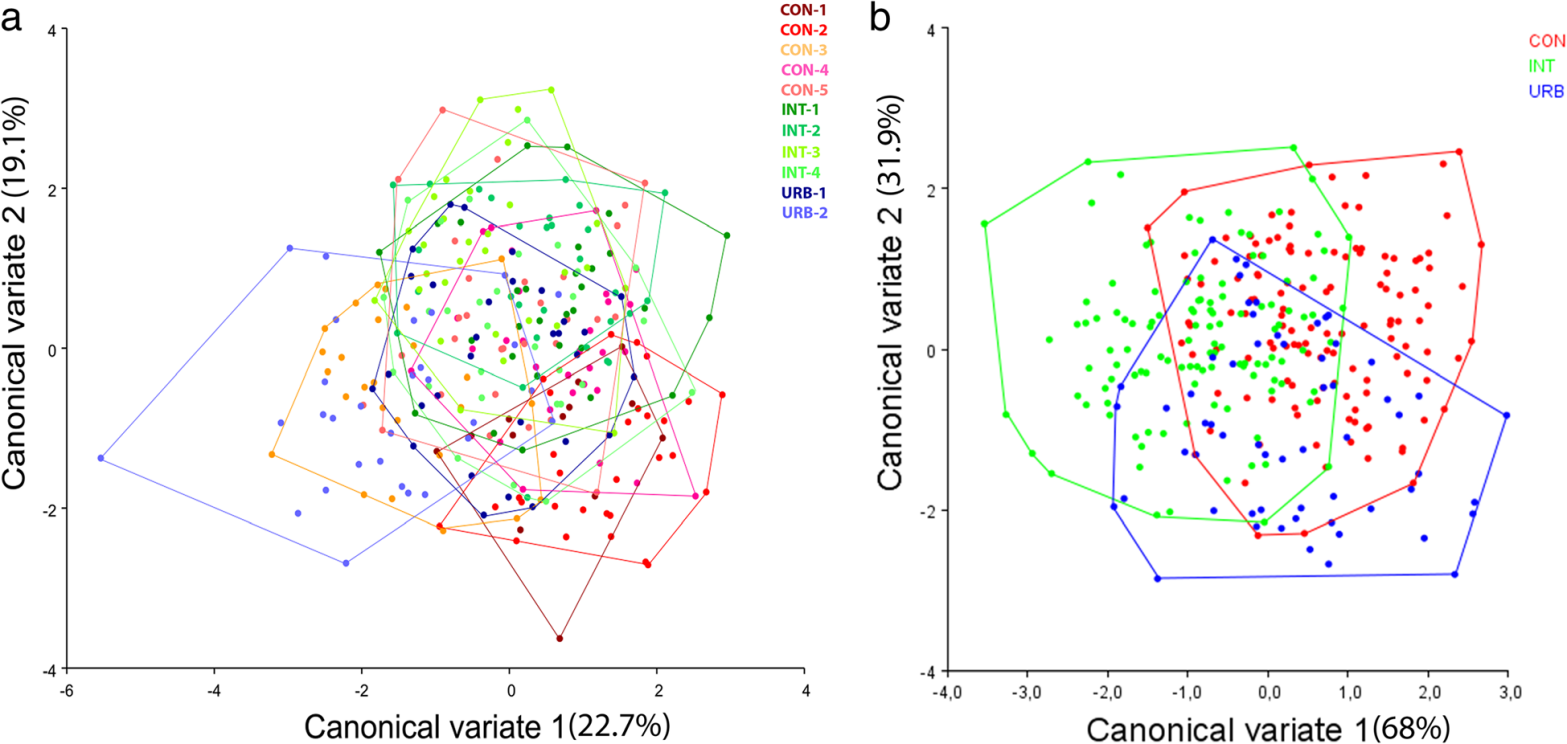
Morphological space produced by CVA of *Aedes aegypti* based on collection sites (**a**) and urban build environments (**b**)

The allometric effect in the populations tested was 9.5% (*P* < 0.0001) and was mathematically excluded from the subsequent analysis. When the populations in each urban build environment (CON, INT and URB) were considered as a metapopulation, wing geometric morphometrics showed a clear pattern of segregation in all analyses. There was a significant correlation between the wing shape variation (Procrustes) and the geographical distance between populations (*r* = 0.44; *r*^*2*^ = 0.20; *P* < 0.001) and the CVA revealed wing-shape variations in the *Ae. aegypti* populations considering both collection sites and population structuring induced by the different levels of urbanization in the collection areas (Fig. 2).

Cross-validated reclassification of the eleven populations yielded significantly high scores. Of 110 comparisons (*P* < 0.0001) only 6 had accuracies of less than 50%, and 15 had accuracies of more than 80%, indicating that there are significant differences in the wing shapes of these populations. The CON-2 population had significantly higher reclassification values (more than 80% reclassification accuracy) than all the other populations apart from URB-1, for which the reclassification score was 66%. In contrast, the CON-1 population had the lowest reclassification scores, with four comparisons yielding scores of less than 50% (Table 2).

**Table 2:** Results of pairwise cross-validated species reclassification (%) based on collection site. Values below the diagonal correspond to mosquitoes from group 1 compared with group 2 and correctly identified; values above the diagonal correspond to mosquitoes from group 2 compared with group 1 and correctly identified. *P*-value (parametric) < 0.0001

		Group 2										
		CON-1	CON-2	CON-3	CON-4	CON-5	INT-1	INT-2	INT-3	INT-4	URB-1	URB-2
Group 1	CON-1	–	90	69	66	66	63	68	76	65	76	66
	CON-2	84	–	73	66	83	83	72	83	62	66	80
	CON-3	61	70	–	66	66	76	65	80	68	63	60
	CON-4	61	83	73	–	63	76	65	76	55	60	73
	CON-5	46	90	69	60	–	80	68	70	79	60	63
	INT-1	46	86	73	66	73	–	55	73	48	70	70
	INT-2	53	80	65	60	70	73	–	63	65	56	73
	INT-3	76	93	88	66	63	70	65	–	58	70	70
	INT-4	46	80	65	60	63	50	62	56	–	53	56
	URB-1	38	66	73	63	56	63	51	70	48	–	63
	URB-2	69	86	76	76	63	63	75	56	65	76	–

The reclassification scores based on urban build environments ranged from 56% (URB *vs* CON) to 70% (INT *vs* CON), with an average of 64% indicating significant differences between metapopulations according to the urban build environment in which they had been collected (Table 3).

**Table 3:** Results of pairwise cross-validated species reclassification (%) for the three urban areas. Values below the diagonal correspond to mosquitoes from group 1 compared with those from group 2 and correctly identified; values above the diagonal correspond to mosquitoes from group 2 compared with group 1 and correctly identified. *P*-value (parametric) < 0.0001

		Group 2		
		CON	INT	URB
Group 1	CON	–	70	67
	INT	69	–	66
	URB	56	58	–

**Fig. 3:**
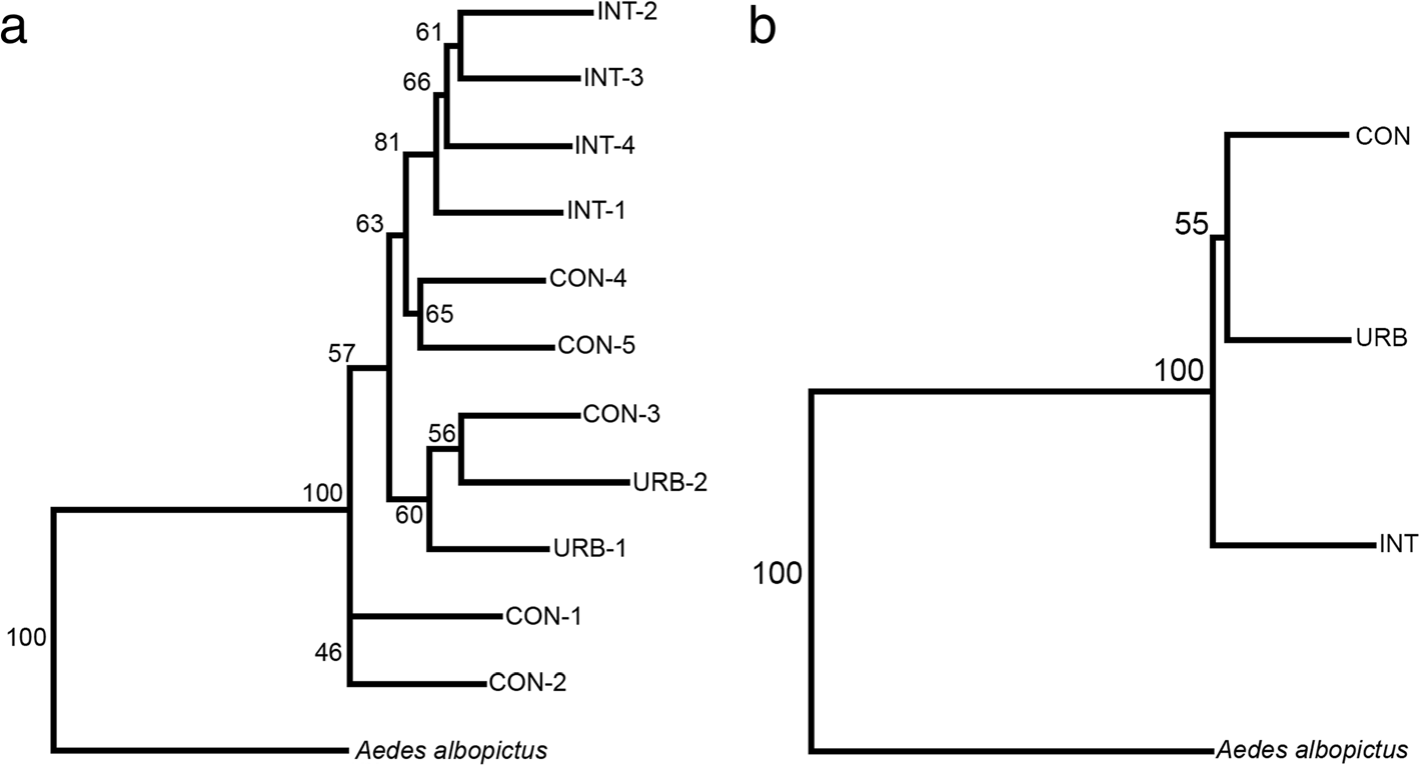
Neighbor-joining trees based on Mahalanobis distance with 1000 bootstrap replicates. **a** For collection sites. **b** For urban build environment

The NJ tree for all the populations shows that the INT populations segregated into a distinct branch, while there was partial overlap between the CON and URB populations (*P* < 0.005) (Fig. 3a). The CON-1 and CON-2 populations segregated into a single branch with 100 bootstrap value, indicating a high level of dissimilarity to the other populations. A second branch contained both populations from the most urbanized area, URB-1 and URB-2, and the CON-3 population. Furthermore, the subsequent analysis of metapopulations showed similar results, with INT segregated in a single branch with 100 bootstrap value (Fig. 3b). The complete segregation of the INT population found in the NJ analysis corroborates previous analyses and highlights the fact that they are dissimilar to the other populations.

**Fig. 4:**
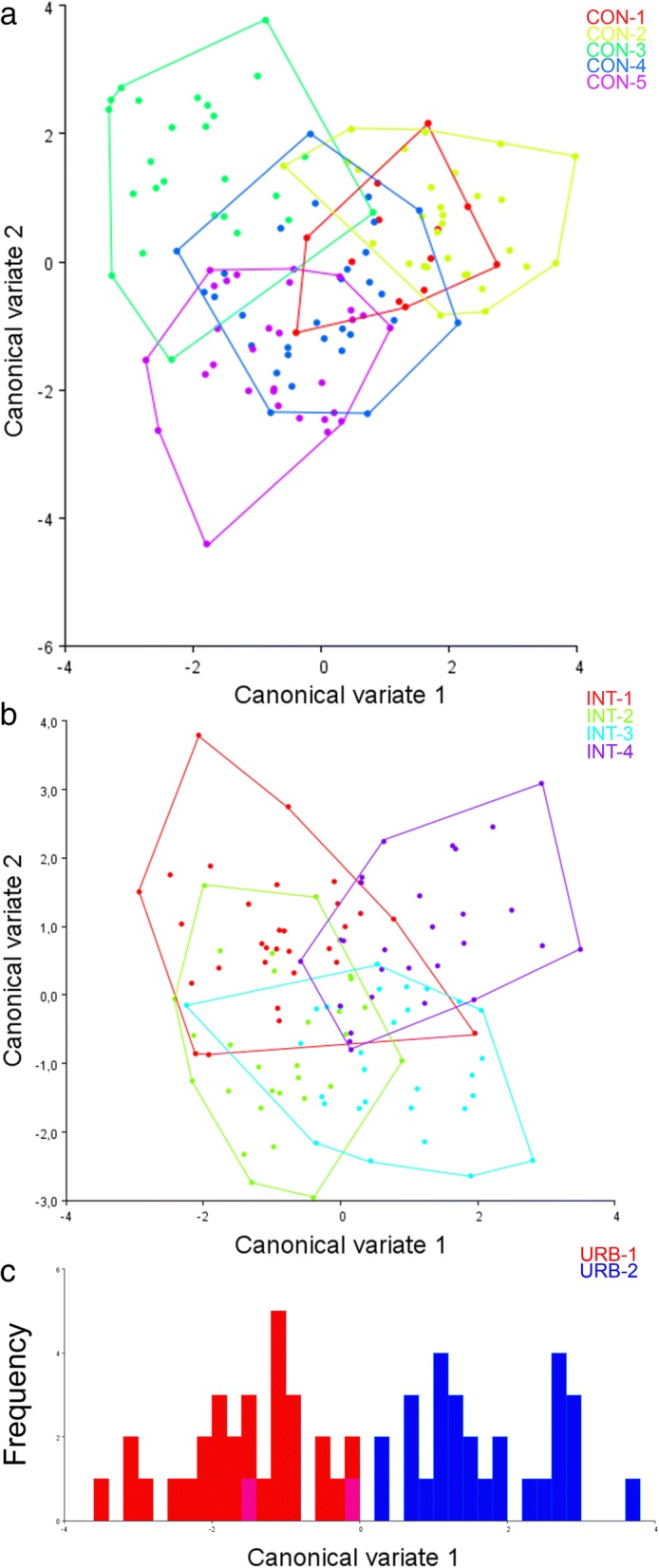
Morphological space produced by CVA of wing shape of *Aedes aegypti* from the CON (**a**) and INT (**b**) areas and wing-shape diagram of first canonical variable for *Aedes aegypti* from the URB area (**c**)

Subsequent analysis considering the variation in populations from each urban build environment individually revealed significant dissimilarity between the CON populations, as shown by the complete segregation between CON-2 and CON-5 and the significant partial segregation between CON-1 and CON-3, CON-1 and CON-5, and CON-2 and CON-3 in the CVA analysis (Fig. 4a). The INT populations occupied the largest area in the morphospace, displaying significant wing shape variations despite their proximity (Fig. 4b). The URB populations were almost completely segregated, as seen in the wing-shape diagram of the first canonical variable where only two wing shapes overlap, indicating a high degree of shape singularities despite the fact that they were collected in areas no more than 1 km apart (Fig. 4c).

**Fig. 5:**
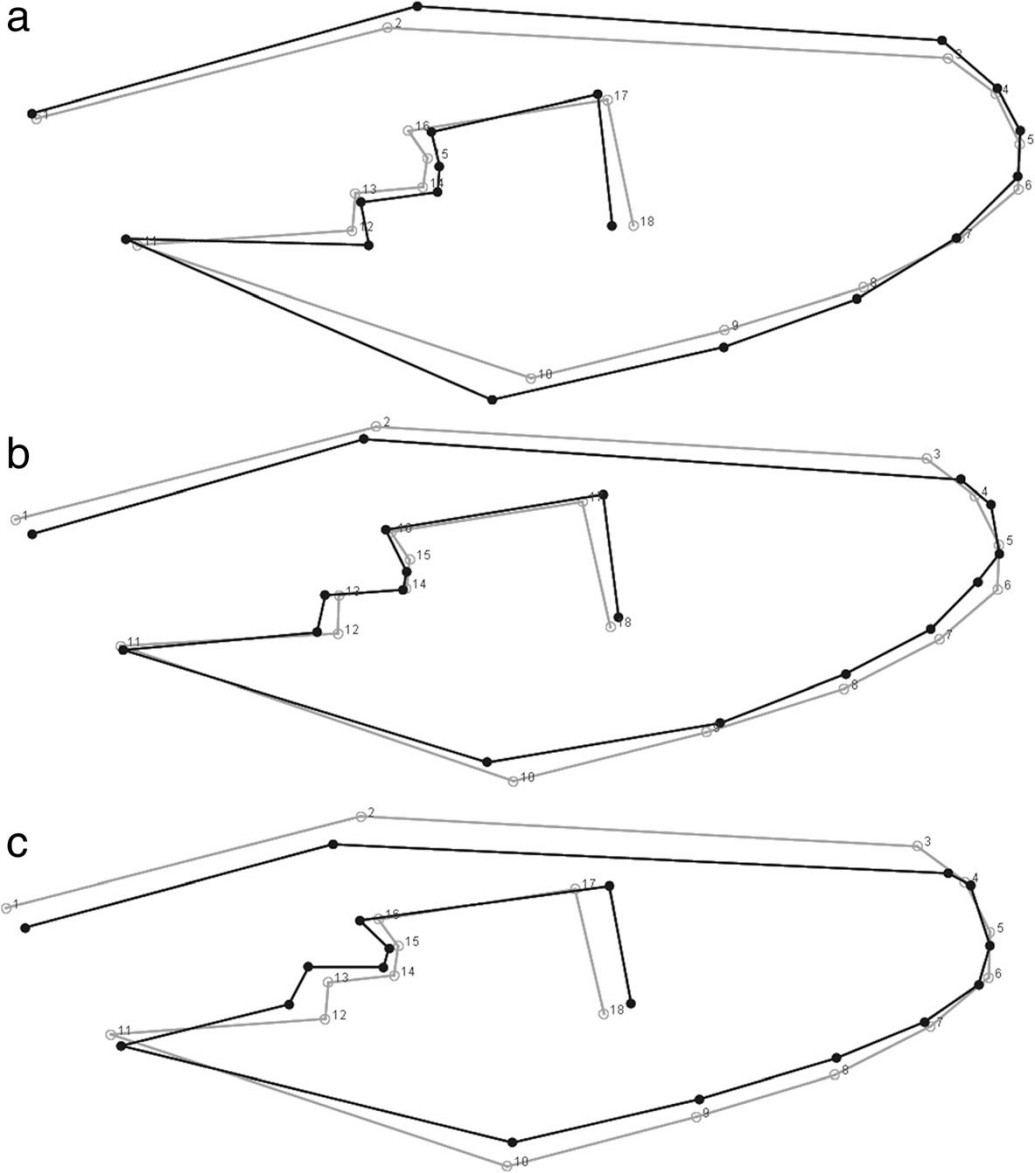
Wireframe representation of shape variations between the three metapopulations. **a** CON *vs* INT; **b** CON *vs* URB; **c** URB *vs* INT

Wing-shape variation within each metapopulation was also compared with the aid of a thin-plate spline and revealed that the INT metapopulation had the greatest variation in wing shape. The greatest variation between metapopulations was between URB and INT and between CON and INT, while the smallest was between CON and URB (Fig. 5).

## Discussion

*Aedes aegypti* is an urban mosquito that is highly adapted to man-made changes and can be found in a wide range of urbanized environments. To thrive in these environments, it must cope with the intense selective pressures such changes bring about. This is reflected in our results, suggesting that different levels of urbanization may have been modulating the population dynamics of *Ae. aegypti* mosquitoes, agreeing with a previous study that used microsatellite markers to analyze *Ae. aegypti* populations from the same collection sites used in this study [39].

Populations from the environments with an intermediate level of urbanization had a significantly wider variety of wing shapes than the other populations. This phenomenon may be explained by the environmental heterogeneity in these areas, which have features of conserved and urbanized environments and a greater range of selective pressures, reflected in a wider variety of wing shapes. This hypothesis is supported by the results of the metapopulation analysis and the analysis of all the study populations individually. In contrast, environments with higher levels of urbanization exert stronger selective pressures, imposing a greater thermal amplitude variation resulting from the so-called “heat islands” [18], fewer resting places and availability of sugar sources, as well as pollution resulting from human action and mechanical removal of breeding sites. In addition, the frequent chemical interventions during control campaigns also account for the population structuring [39].

However, rather than increasing wing-shape variability, these stronger selective pressures increase the rate at which changes in wing shape occur, corroborating the findings of Louise et al. [22]. On the other hand, populations in conserved areas showed moderate wing-shape variation, which may be explained by the inherently more complex habitats with higher levels of biodiversity and density-dependent pressures [40–42]. This hypothesis is corroborated by a previous study for the same geographical region, in which the drivers for species richness and composition were found to be associated with urbanization processes [43].

The results of the cross-validated reclassification indicate a very significant variation in wing shape between the populations. Of 110 possible population comparisons, cross-validated reclassification scores for 15 were above 80% and only 6 below 50%. Considering that these populations belong to a microgeographical region, these results indicate significant population structuring in *Ae. aegypti* as a result of urbanization processes.

Uncovering the mechanisms by which urbanization processes interact with mosquito populations, especially on a small scale, is of great epidemiological importance, particularly in the case of *Ae. aegypti*. A previous study conducted by Araujo et al. [44] found that urban heat islands have a higher incidence of dengue than other urban areas, indicating that variations in urbanization may be modulating dengue transmission. Similar findings were reported in a study by Lambrechts et al. [45], who found that temperature variations may influence dengue transmission patterns.

## Conclusions

Areas with different levels of urbanization have different features and selective pressures. These pressures act on *Ae. aegypti*, modulating the population structuring in this species. Furthermore, the fact that *Ae. aegypti* thrives in the urban environment, which provides a virtually unlimited number of breeding sites and human hosts for blood-feeding, may lessen the need for this species to migrate to new areas in search of food and breeding sites [46–48]. Finally, variations in environmental features, such as those caused by urbanization, appear to play an important role in the dynamics of *Ae. aegypti* populations. Wing geometric morphometrics was successfully used in this study to glimpse microevolutionary processes on *Ae. aegypti* populations in urban areas.

## Additional files

Additional file 1: **Table S1.** Collection of immature *Aedes aegypti* according to breeding site. (DOCX 14 kb)

Additional file 2: **Figure S1.** UPGMA tree based on geographical distance (km) for all populations. (TIF 1126 kb)

## Abbreviations

CON-1: Anhanguera Park
CVA: Canonical Variate Analysis
INT-2: Communication and Art School
CON: conserved areas
CON-2: Eucalipto Park
CON-3: Independência Park
INT: Intermediate areas
URB-2: Medicine School
NJ: Neighbor-joining
INT-3: Physics Institute
CON-4: Piqueri Park
CON-5: Previdência Park
URB-1: Public Health School
INT-1: University of São Paulo Student Accommodation
URB: urbanized areas
INT-4: Veterinary School
