# Supplementary material for: Wing morphometric variability in Aedes aegypti (Diptera: Culicidae) from different urban built environments
Source: Parasit Vectors. 2018 Oct 26;11:561. doi: 10.1186/s13071-018-3154-4 (PMC6203966; doi:10.1186/s13071-018-3154-4)
Supplement: Supplementary file 1 — Table S1. Collection of immature Aedes aegypti according to breeding site. (DOCX 14 kb) [file 13071_2018_3154_MOESM1_ESM.docx]

Table S1. Collection of immature *Aedes aegypti* according to breeding site.

| **Collection Site** | **Collection Tool** | Bamboo | Epiphytic bromeliad | Terrestrial bromeliad | Tree hole | Ovitrap container | Artificial container* | **Total of Larvae** |
| --- | --- | --- | --- | --- | --- | --- | --- | --- |
| Anhanguera Park | Suction | x |  | x |  |  |  | 40 |
|  | Dipper | x |  | x |  |  | x | 9 |
| Eucalipto Park | Suction | x |  |  |  |  |  | 192 |
| Independência Park | Emptying container |  |  |  |  |  | x | 59 |
| Piqueri Park | Suction | x | x | x | x |  | x | 365 |
|  | Dipper |  |  |  |  |  | x | 16 |
|  | Emptying container |  |  |  |  |  | x | 3 |
| University of São Paulo Student Accommodation | Ovitraps |  |  |  |  | x |  | 38 |
| Communication and Art School | Ovitrap |  |  |  |  | x |  | 43 |
| Physics Institute | Ovitrap |  |  |  |  | x |  | 43 |
| Veterinary School | Ovitrap |  |  |  |  | x |  | 36 |
| Public Health School | Ovitrap |  |  |  |  | x |  | 95 |
| Medicine School | Suction |  |  |  |  |  | x | 36 |
|  | Ovitrap |  |  |  |  | x |  | 89 |
| **Total of specimens** |  |  |  |  |  |  |  | **1,064** |

*Artificial containers (cans, bottles, plastic gallon, tires, water tank).
