# Supplementary figures and images for: Wing morphometric variability in Aedes aegypti (Diptera: Culicidae) from different urban built environments
Source: Parasit Vectors. 2018 Oct 26;11:561. doi: 10.1186/s13071-018-3154-4 (PMC6203966; doi:10.1186/s13071-018-3154-4)

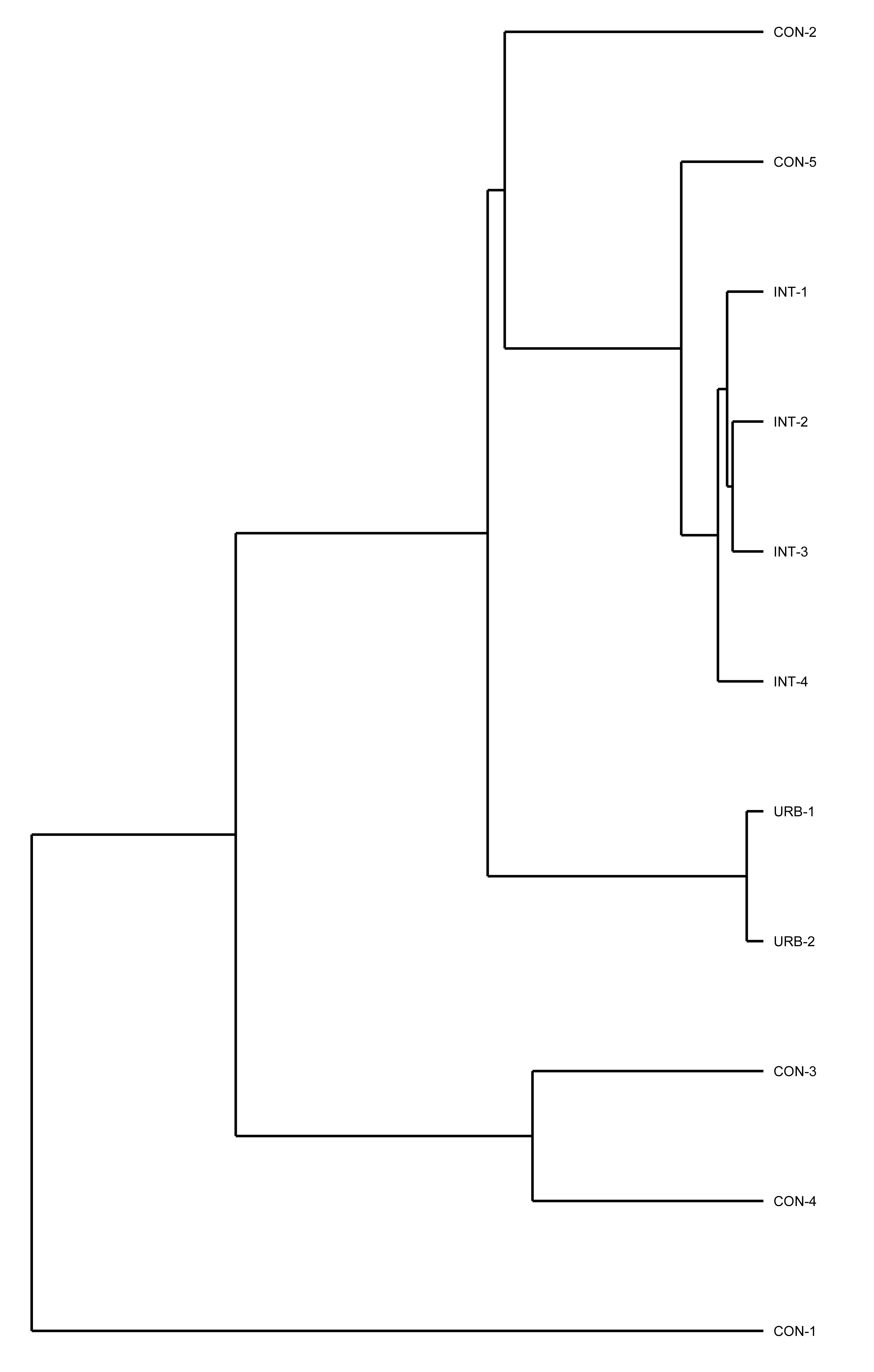

Supplement: Supplementary file 2 — Figure S1. UPGMA tree based on geographical distance (km) for all populations. (TIF 1126 kb) [file 13071_2018_3154_MOESM2_ESM.tif]
